# Identification of an Individualized Immune-Related Prognostic Risk Score in Lung Squamous Cell Cancer

**DOI:** 10.3389/fonc.2021.546455

**Published:** 2021-03-03

**Authors:** Yuan Zhuang, Sihan Li, Chang Liu, Guang Li

**Affiliations:** Department of Radiation Oncology, The First Affiliated Hospital of China Medical University, Shenyang, China

**Keywords:** immune-related genes, prognosis, lung squamous cell carcinoma (LUSC), TCGA, transcriptome

## Abstract

**Background:** Lung squamous cell carcinoma (LUSC) is one of the most common histological subtypes of non-small cell lung cancer (NSCLC), and its morbidity and mortality are steadily increasing. The purpose of this study was to study the relationship between the immune-related gene (IRGs) profile and the outcome of LUSC in patients by analyzing datasets from The Cancer Genome Atlas (TCGA).

**Methods:** We obtained publicly available LUSC RNA expression data and clinical survival data from The Cancer Genome Atlas (TCGA), and filtered IRGs based on The ImmPort database. Then, we identified risk immune-related genes (r-IRGs) for model construction using Cox regression analysis and defined the risk score in this model as the immune gene risk index (IRI). Multivariate analysis was used to verify the independent prognostic value of IRI and its association with other clinicopathological features. Pearson correlation analysis was used to explore the molecular mechanism affecting the expression of IRGs and the correlation between IRI and immune cell infiltration.

**Results:** We screened 15 r-IRGs for constructing the risk model. The median value of IRI stratified the patients and there were significant survival differences between the two groups (*p* = 4.271E-06). IRI was confirmed to be an independent prognostic factor (*p* < 0.001) and had a close correlation with the patients' age (*p* < 0.05). Interestingly, the infiltration of neutrophils or dendritic cells was strongly upregulated in the high-IRI groups (*p* < 0.05). Furthermore, by investigating differential transcription factors (TFs) and functional enrichment analysis, we explored potential mechanisms that may affect IRGs expression in tumor cells.

**Conclusion:** In short, this study used 15 IRGs to build an effective risk prediction model, and demonstrated the significance of IRGs-based personalized immune scores in LUSC prognosis.

## Introduction

Lung squamous cell carcinoma (LUSC) is one of the most common subtypes of non-small cell cancer (NSCLC), which is based primarily on tobacco exposure (1). Compared with lung adenocarcinoma (LUAD), LUSC has a poor clinical prognosis and limited treatment progress. Due to the lack of available targeted agents, traditional platinum-based chemotherapy remains the main therapeutic regimen for LUSC (2). However, the emergence of drug resistance has compelled more optimized remedies. Recently, immunotherapy has shown encouraging treatment results in some cancers including NSCLC (3, 4). And thus far, pembrolizumab combined with platinum-based chemotherapy has been extended as the first-line therapy to LUSC patients with a PD-L1 tumor proportion score (TPS) of 1% or greater and without a sensitizing EGFR mutation or ALK translocation (5, 6). Furthermore, some PD-1/PD-L1 blockades, including nivolumab, pembrolizumab, and atezolizumab have been approved as the second-line settings for advanced patients (7).

Biomarkers, especially gene expression in tumor tissues, can reliably predict disease prognosis and patients' survival, which are tremendously valuable to advise rational clinical treatment of cancer (8). It is necessary to classify the subgroup of patients with a high risk of relapse and to give them targeted surveillance and other systemic therapies. The existence of large-scale public cohorts and sound gene expression databases has laid the foundation for identifying prognostic biomarkers (9, 10). Various components of the immune system have been considered as determinants of the occurrence and progress of cancer (11, 12). Therefore, studying the differential expression of immune-related genes (IRGs) in tumor tissue samples is of great significance for exploring the tumor microenvironment, improving clinical diagnosis, optimizing immunotherapy, and assessing the prognosis of patients (13).

Li et al. thoroughly explored the prognostic value of IRGs in patients with non-squamous non-small cell lung cancer (14). However, the clinical relevance and prognostic value of IRGs in lung squamous cell carcinoma (LUSC) remain unknown. In this study, we integrated the IRGs' expression profiles with clinical information to build and validate an individualized prognostic index for LUSC. We systematically analyzed the potential clinical utility of IRGs in prognostic stratification and their enlightening significance as a composite index to guide the treatment of LUSC, which could establish a foundation for subsequent immunotherapy and increase the predicted accuracy for overall survival (OS).

## Materials and Methods

### Data Collection

Transcriptome RNA-sequencing data and the corresponding clinical follow-up information with respect to lung squamous cell carcinoma patients were downloaded from the Genomic Data Commons Data Portal of TCGA (https://portal.gdc.cancer.gov/). The expression data was obtained via HTSeq-FPKM and contained 502 LUSC tissues and 49 adjacent non-tumorous tissue samples as of February 2020. Next, immune-associated genes were obtained from the immunology database and analysis portal (ImmPort) (https://www.immport.org/shared/home).

### Differential Gene Analysis and Filtering

Differential gene expression was investigated using the edgeR package, and the parameters log fold change (FC)>2 or < -2, and false discovery rate (FDR) < 0.05. Then, differential IRGs were filtrated from all of the differentially expressed genes. Moreover, univariate Cox regression analysis was applied to opt for the IRGs associated with the survival time, using the R software “survival” package.

### Functional Enrichment Analysis

Functional enrichment was investigated using Gene Ontology (GO) and Kyoto Encyclopedia of Genes and Genomes (KEGG) analyses to explore the potential functions of IRGs. The false discovery rate (FDR) was set as < 0.05 as the threshold.

### Exploration of the Molecular Mechanism of Immune Genes

It is well-known that transcription factors (TFs) play a key role in the degree of gene expression. Here, we performed a correlation analysis between prognosis-related IRGs and differential TFs. The list of 318 (TFs) was downloaded from the Cistrome Cancer database (http://www.cistrome.org/). For the analysis, the corFilter was set to 0.4 and the *P*-value was set to 0.001. Additionally, we also obtained the regulatory network of these IRGs and TFs, using the Cytoscape software version 3.7.2.

### Construction and Validation of the IRGs-Based Prognostic Risk Model

Multivariate Cox regression analysis was performed on the IRGs; the weighted score was calculated as the risk score for each patient. The score of the predictive model was determined as = (exprIRG_1_×coef_1_) + (exprIRG_2_×coef_2_) +. + (exprIRG_n_×coef_n_). According to the median risk score as the cut-off value, LUSC patients were divided into the low-risk group and high-risk group. The “survival” and “survminer” R packages were used for Kaplan-Meier analysis and the visualization of the survival curves. Furthermore, a ROC curve was plotted and the area under the curve (AUC) was calculated to verify the predictive power of this risk model; the “survivalROC” R package was used. Subsequently, following the trend of risk scores, the patients' survival status and the expression of the IRGs involved in this model were observed. Univariate and multivariate analyses were further performed to understand whether the risk score could be an independent prognostic predictor. The relationship between the expression of IRGs and other clinicopathological factors was also investigated.

### Correlation Analysis of Immune Cells Infiltration

The TIMER database (https://cistrome.shinyapps.io/timer/) was used to evaluate the abundance of tumor-infiltrating immune cells, including B cells, CD4 T cells, CD8 T cells, macrophages, neutrophils, and dendritic cells. Briefly, we downloaded the immune infiltration level of LUSC patients using the TIMER online tool and calculated the correlation between the immune gene risk score and each type of infiltrating immune cells.

### Statistical Analysis

All analyses were conducted using R software (version 3.6.2). The differential expression of genes in the TCGA cohorts was evaluated by the Wilcox test. Univariate and multivariate analyses were performed via Cox regression. Survival analysis was performed using the log-rank test. The correlation was performed by Pearson correlation analysis. Differences among clinicopathologic features were tested by Student's *t*-tests. *P* values < 0.05 were considered significant in all statistical tests.

## Results

### Screening and Identification of IRGs

**Figure 1:**
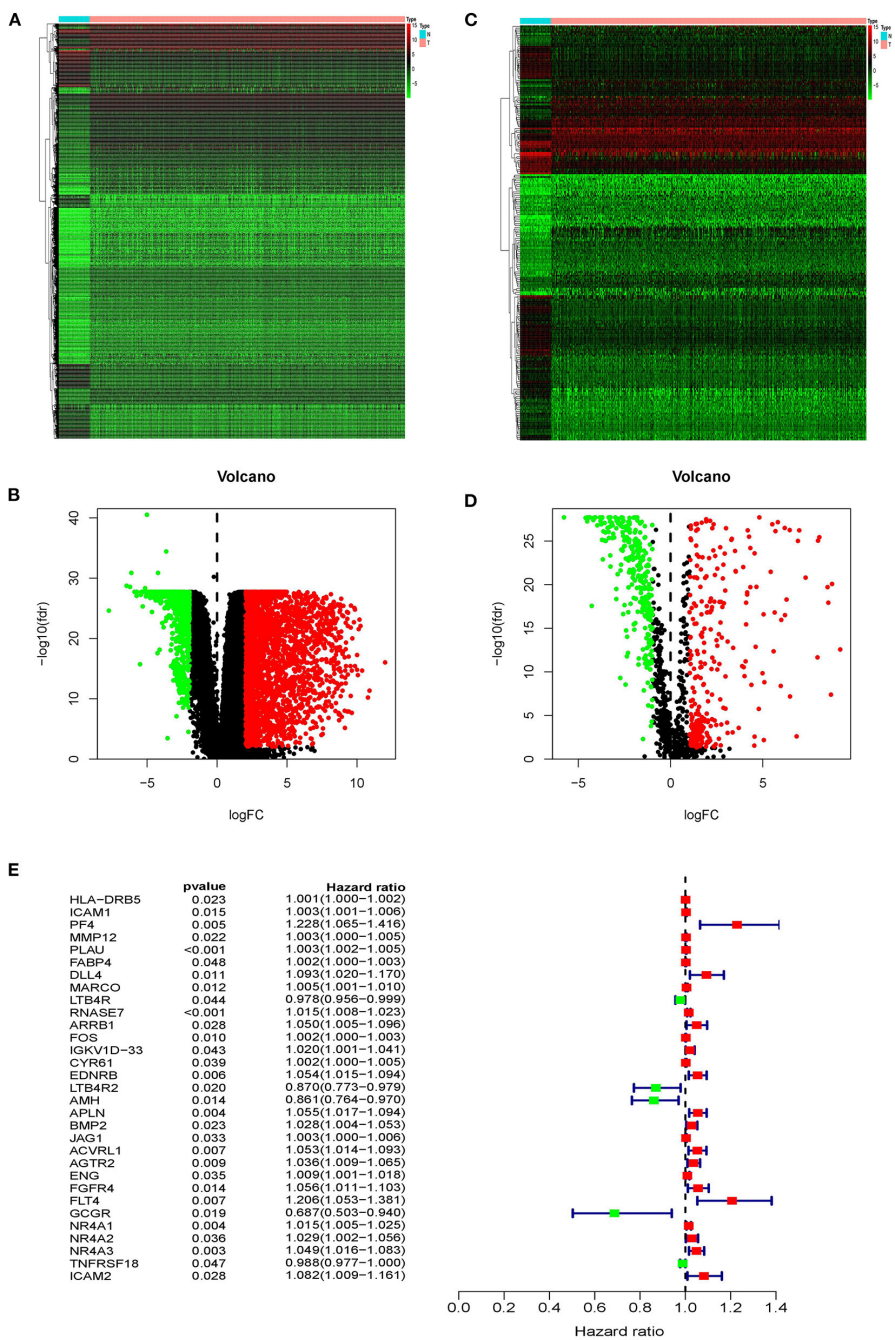
Differentially expressed immune-related genes. Heatmap **(A)** and volcano plot **(B)** of differentially expressed genes between lung squamous cell carcinoma (LUSC) and adjacent non-tumor tissues. Heatmap **(C)** and volcano plot **(D)** demonstrating differentially expressed immune-related genes (IRGs). The black dots represent the undifferentiated genes, while the red and green dots represent the differentiated genes. **(E)** Forest plot of hazard ratios showing the prognostic values of immune-related genes.

Based on the TCGA database, we compared gene expression in tumor tissues and non-tumorous tissues in LUSC transcriptome RNA data and screened 3599 differential genes, of which 2604 genes were up-regulated in tumors and 995 genes were down-regulated in tumors (Figures 1A,B). Later, downloading IRGs from the ImmPort database and performing a differential analysis revealed that 223 candidate genes were differentially expressed between the tumor and the adjacent tumor, of which 110 genes were up-regulated in the tumor and 113 genes were down-regulated (Figures 1C,D). The survival time and survival status are the chief clinical outcomes of patients. By integrating the differential immune gene matrix and the clinical outcome matrix, 31 prognosis-associated IRGs (p-IRGs) were obtained, using univariate Cox regression analysis (Figure 1E).

### Function Enrichment Analysis

**Figure 2:**
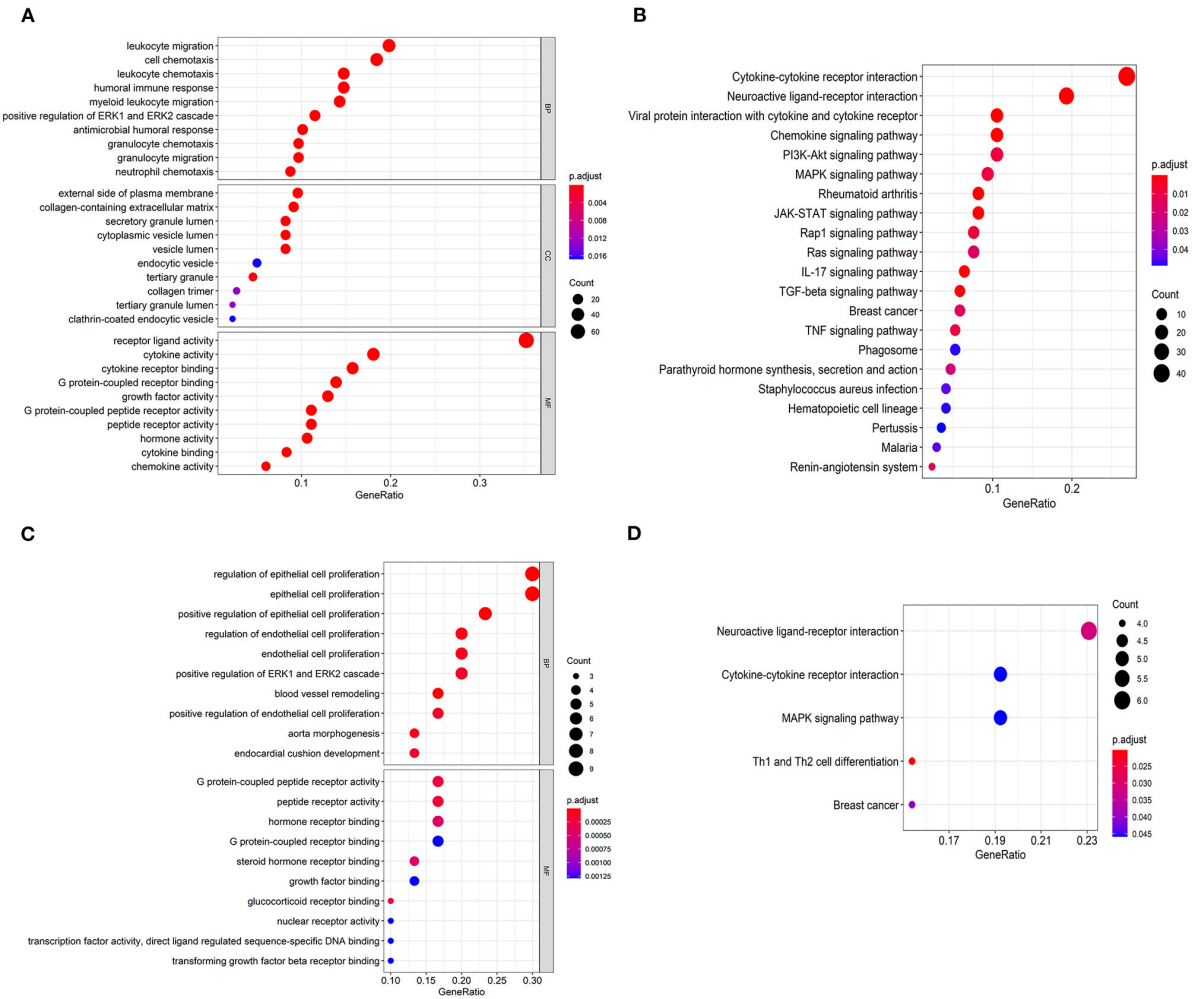
Gene functional enrichment of IRGs. Gene ontology analysis (GO) of differentially expressed immune-related genes **(A)** and the significant Kyoto Encyclopedia of Genes and Genomes pathways (KEGG) **(B)**. Gene ontology analysis (GO) of prognostic-associated immune-related genes **(C)** and the most significant Kyoto Encyclopedia of Genes and Genomes pathways (KEGG) **(D)**.

As far as we know, Gene Ontology (GO) is divided into three parts: Molecular Function (MF), Biological Process (BP), and Cellular Component (CC) (15). We performed functional enrichment analysis on 223 differentially expressed IRGs and identified 1,245 GO terms and 21 important KEGG pathways. The results showed that the eight main GO terms were: receptor-ligand activity, leukocyte migration, cell chemotaxis, cytokine activity, cytokine receptor binding, humoral immune response, leukocyte chemotaxis, and myeloid receptor (Figure 2A). Furthermore, the top six KEGG pathways were Cytokine-cytokine receptor interaction, Neuroactive ligand-receptor interaction, Viral protein interaction with cytokine and cytokine receptor, Chemokine signaling pathway, PI3K-Akt signaling pathway, and MAPK signaling pathway (Figure 2B). Unsurprisingly, gene function enrichment analysis showed that inflammation-related pathways were the most common. Next, we performed similar enrichment analysis on p-IRGs and found that these p-IRGs were most abundant in terms of GO terms related to biochemical processes (Figure 2C). Moreover, the Neuroactive ligand-receptor, Cytokine-cytokine receptor interaction, and MAPK signaling pathway were the most commonly identified KEGG pathways (Figure 2D).

### Association Between Differential IRGs and Transcription Factors

**Figure 3:**
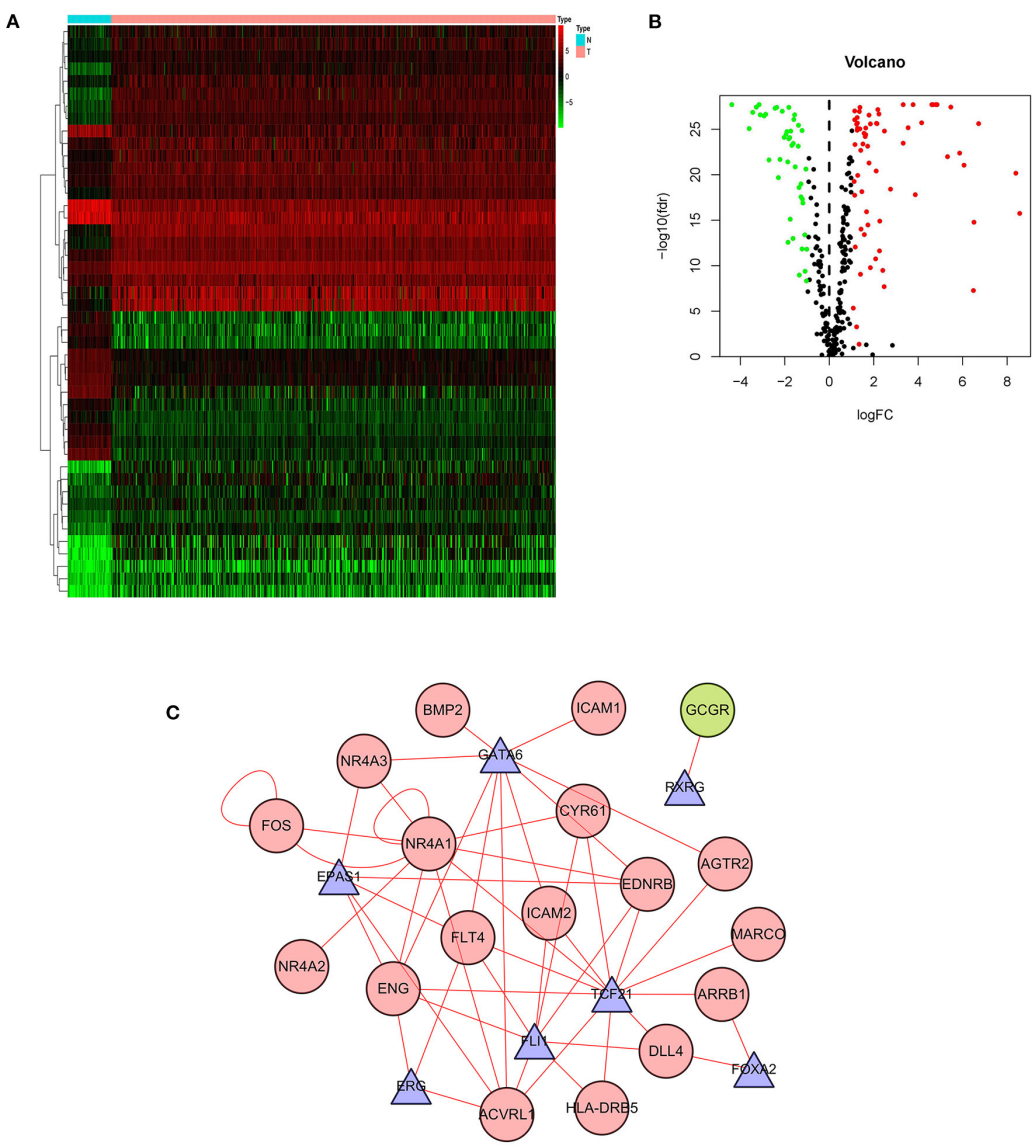
Transcription factor-mediated regulatory network. Heatmap **(A)** and volcano plot **(B)** showing differentially expressed transcription factors (TFs). **(C)** Regulatory network based on clinically relevant TFs and IRGs.

Gene expression is regulated by transcription factors (TFs). Therefore, we explored the regulatory relationship between IRGs and TFs to explore the possible molecular mechanisms affecting p-IRGs expression. First of all, we isolated 46 out of the 318 transcription factors with differential expression in tumors and adjacent tissues (Figures 3A,B). According to the correlation analysis of p-IRGs and TFs, it can be concluded that 9 TFs, including EPAS1, ERG, FLI1, FOXA2, FOS, GATA6, NR4A1, RXRG, and TCF21 were mutually regulated with 18 immune genes. We then established a regulatory network based on the above results and exhibited this network via Cytoscape software (Figure 3C). Notably, NR4A1 dominated the network. It was a transcription factor of the regulatory nuclear receptor family, which was positively correlated with the expression of NR4A3, NR4A2, FOS, CYR61, EDNRB, and ACVRL1. It was also a prognostic high-risk gene in the model and can be positively regulated by TCF21 and itself.

### Construction of an IRGs Risk Model for LUSC Patients

**Figure 4:**
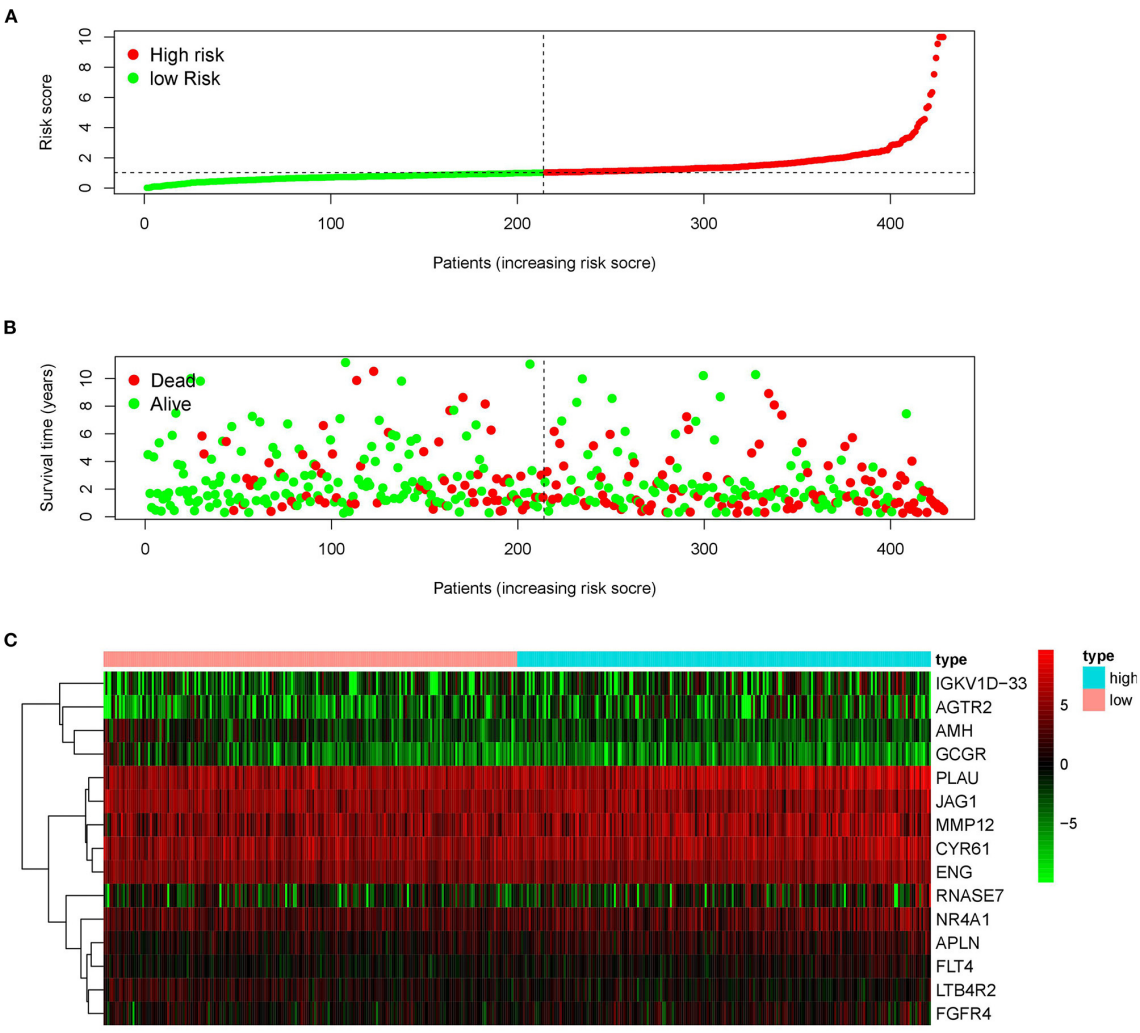
Construction of the prognostic risk model based on immune-related genes. **(A)** Rank of prognostic risk score and distribution of groups. **(B)** Survival status of patients in different groups. **(C)** Heatmap of expression profiles of included genes.

Since the 31 p-IRGs are not the common driver genes for lung squamous cell carcinoma, we speculated that abnormal expression of these IRGs may be a prognostic factor. Next, we put 31 p-IRGs into the multivariate Cox regression analysis and obtained 15 risk-related immune genes (r-IRGs) and their coefficients involved in the construction of the risk model (Table 1). The 15 r-IRGs included MMP12, PLAU, RNASE7, IGKV1D-33, CYR61, LTB4R2, AMH, APLN, JAG1, AGTR2, ENG, FGFR4, FLT4, GCGR, and NR4A1. The risk score in the model was calculated as ∑coefficients ^*^ expression values. Here, we defined this risk score as the “Immune gene Risk Index” (IRI). The median value of IRI was 1.020488, which was set as a cut-off value, and patients were stratified into two groups: high-risk group and low-risk group. The survival status and r-IRGs expression of the two groups were shown in the Figures 4A–C.

**Table 1:** Risk immune-related genes (r-IRGS) names and coefficients in the risk model.

**ID**	**Coef**
MMP12	0.002042
PLAU	0.002818
RNASE7	0.01687
IGKV1D-33	0.022426
CYR61	−0.00331
LTB4R2	−0.18137
AMH	−0.09952
APLN	0.039632
JAG1	0.00371
AGTR2	0.029106
ENG	−0.01417
FGFR4	0.050224
FLT4	0.221289
GCGR	−0.27283
NR4A1	0.019365

### Performance of r-IRGS Risk Model in Predicting the Prognosis of LUSC

**Figure 5:**
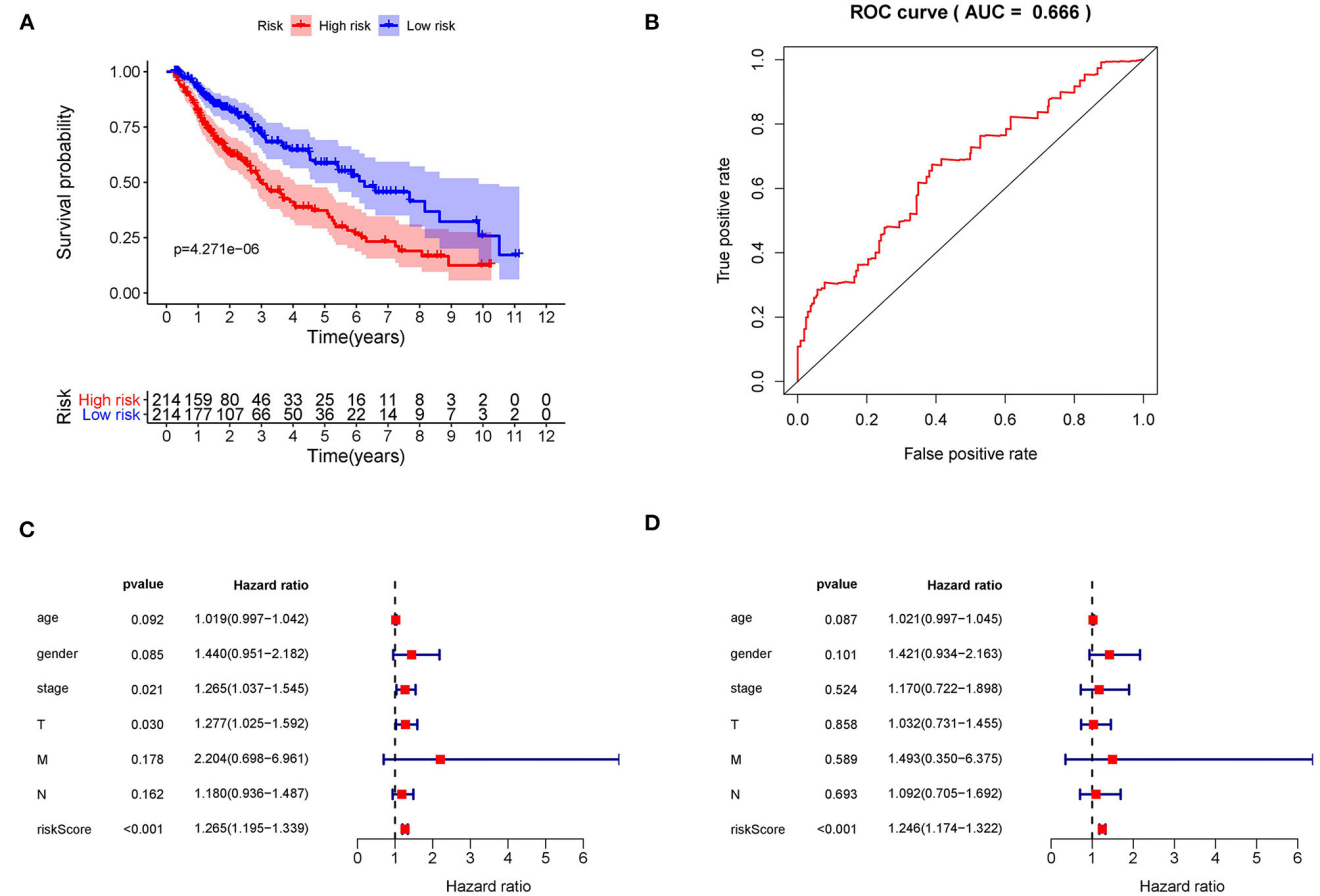
The prognostic value of the risk score. **(A)** The overall survival (OS) time of patients in the high-risk group and low-risk group. **(B)** The ROC curves of OS for the 15-gene immune-related risk score. Forest plot of hazard ratios showing the prognostic values of immune-related genes involved in the risk model based on Univariate analysis **(C)** and multivariate analysis **(D)**.

Kaplan–Meier (KM) survival analysis showed significant differences in overall survival between the high-risk and the low-risk groups (Figure 5A, *p* = 4.271E-06). Subsequently, to validate the reliability of this risk model, we applied ROC analysis to the cohort. The area under the curve (AUC) of the ROC curve is 0.666, indicating that the r-IRGs risk model has a certain degree of applicability for predicting prognosis in patients with LUSC (Figure 5B). Besides, both univariate (Figure 5C, *p*=6.00E-16) and multivariate (Figure 5D, *p* = 3.47E-13) Cox survival analysis indicated that IRI could be used as an independent prognostic factor for lung squamous carcinoma patients' survival (Table 2).

**Table 2:** Univariate and multivariate regression analysis of lung squamous cell carcinoma.

**Variables**	**Univariate analysis**		**Multivariate analysis**	
	**Hazard ratio (95%CI)**	***P-*value**	**Hazard ratio (95%CI)**	***P*-value**
Age	1.019 (0.997–1.042)	0.092	1.021 (0.997–1.045)	0.087
Gender	1.440 (0.951–2.182)	0.085	1.421 (0.934–2.163)	0.101
Stage	1.265 (1.037–1.545)	0.021	1.170 (0.722–1.898)	0.524
T	1.277 (1.025–1.592)	0.030	1.032 (0.731–1.455)	0.858
M	2.204 (0.698–6.961)	0.178	1.493 (0.350–6.375)	0.589
N	1.180 (0.936–1.487)	0.162	1.092 (0.705–1.692)	0.693
IRI	1.265 (1.195–1.339)	<0.001	1.246 (1.174–1.322)	<0.001

### Association Between IRI and Other Clinicopathologic Factors in LUSC

**Figure 6:**
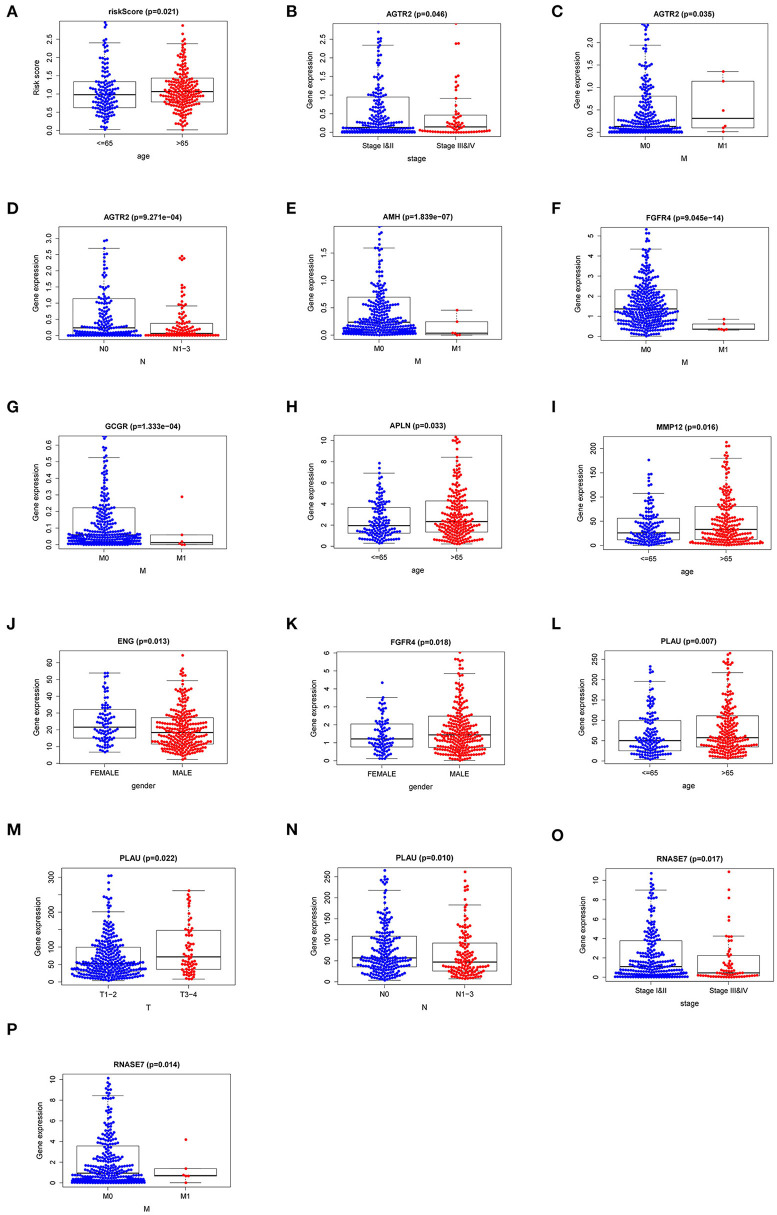
The relationships between the risk immune gene and clinicopathologic characters. **(A)** The correlation between the Immune gene Risk Index (IRI) and patients' age. The relationships between AGTR2 and **(B)** tumor stage; **(C)** distant metastasis; **(D)** lymph node metastasis. The relationships between distant metastasis and **(E)** AMH; **(F)** FGFR4; **(G)** GCCR. The relationships between patients' age and APLN **(H)** and MMP12 **(I)**. The relationships between patients' gender and ENG **(J)** and FGFR4 **(K)**. The relationships between PLAU and **(L)** age; **(M)** T stage; **(N)** lymph node metastasis. The relationships between RNASE7 and **(O)** tumor stage and **(P)** distant metastasis.

To explore the more clinical relevance of IRI and r-IRGs, we analyzed the relationship between them and clinical pathology, including age, gender, American Joint Committee On Cancer (AJCC) stage, tumor burden (T), regional lymph node metastasis (N), and distant metastasis (M). IRI was positively correlated with patient age, but there were no significant differences between other factors (Figure 6A, *p*=0.021). AGTR2 was positively correlated with advanced cases (Figure 6B), distant metastases (Figure 6C), and no lymph node metastases (Figure 6D). AMH (Figure 6E), FGFR4 (Figure 6F), and GCCR (Figure 6G) were correlated with no distant metastasis. APLN (Figure 6H) and MMP12 (Figure 6I) were also associated with older age. ENG (Figure 6J) was higher expressed in male patients, while FGFR4 (Figure 6K) was higher expressed in female patients. PLAU was positively correlated with the elderly (Figure 6L), advanced T-stage cases (Figure 6M), and cases without lymph node metastasis (Figure 6N). However, RNASE7 was negatively correlated with advanced cases (Figure 6O) and distant metastatic cases (Figure 6P).

### Correlation Between IRI and Immune Cell Infiltration

**Figure 7:**
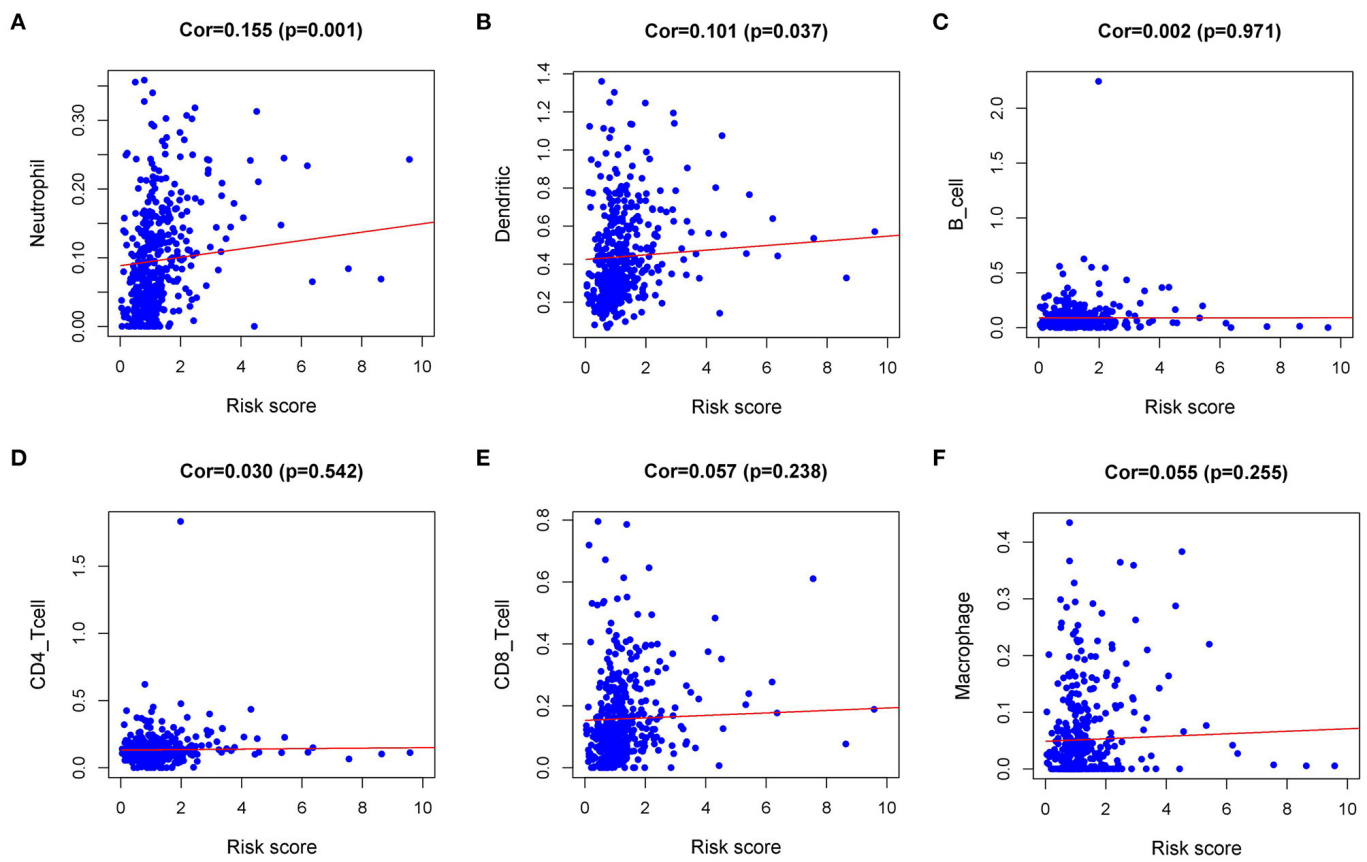
Relationships between the Immune gene Risk Index (IRI) and infiltration abundances of immune cells. **(A)** neutrophils; **(B)** dendritic cells; **(C)** B cells; **(D)** CD4 T cells; **(E)** CD8 T cells; and **(F)** macrophages.

Immune infiltration in the tumor microenvironment (TME) is necessary to study the interaction between tumors and immunity (16). To investigate the capability of IRI to predict the state of the immune microenvironment, we explored the correlation between IRI and immune cell infiltration. Our analysis indicated that the IRI was significantly positively correlated to the infiltration of neutrophils and dendritic cells (Figures 7A,B). However, there was no statistical significance in the correlation between IRI and the abundance of the other four types of immune cells (Figures 7C–F).

## Discussion

It is indispensable to search for reliable prognostic biomarkers that can identify patients with a higher risk of poor survival. There are a lot of researches on the prognosis-related biomarkers of lung squamous cell carcinoma, but the applicability and accuracy of these indicators are not satisfactory (17). So far, clinical guidelines are still based on tumor staging (AJCC/UICC-TNM classification), which contains data on tumor burden (T), the presence of cancer cells in draining and regional lymph nodes (N), and evidence of metastasis (M) to assess disease stage and predict survival (18). With the exciting development of tumor immunotherapy, the role of tumor-associated immunology in the development of cancer has once again been taken into consideration. Galon et al. first proposed a concept of immune scores, and then the immune score was described as a prognostic marker for patients with early colorectal cancer (19–21). Recently, several studies have established a risk model based on immune-related genes (IRGs) to distinguish patients with different risk levels, which enables patients to receive more optimized and rational treatment. Such studies include colorectal cancer (22, 23), breast cancer (24, 25), head and neck squamous cell cancer (26), thyroid cancer (13), and so on. Based on the TCGA-LUSC dataset, we developed a prognostic risk model that incorporates 15 r-IRGs selected according to multivariate regression analysis. We also explored the relevance of this model score to clinicopathological features and immune cell infiltration in the immune microenvironment. Furthermore, the potential molecular mechanisms that affect immune gene expression were discussed.

The characteristics of tumor development depend on a succession of alterations in the genome (27). We concentrated our research on the alterations of the immunogenomic profiles (28). Massive RNA-sequencing weighted immune-related gene expression can reflect the status of tumor cells. In KEGG analysis, the most significant pathway was enriched in the Cytokine-cytokine receptor interaction process. In terms of biological process and molecular function, the top enriched GO terms were highly related to receptor-ligand activity and leukocyte migration. As expected, the functional enrichment analysis showed that these genes were indeed primarily involved in immune-related signaling pathways. Additionally, these enriched signaling pathways frequently participate in tumorigenesis driving, invasion, and metastasis, thus inferring that immune-related genes have potential value in predicting clinical outcomes.

To explore potential molecular mechanisms, we constructed a TF-mediated network to uncover the significant TFs regulating the IRGs in this risk model. NR4A1 was dominant in the network and positively correlated with the expression of NR4A3, NR4A2, FOS, CYR61, EDNRB, and ACVRL1. Recently, it was also found that NR4A1 is associated with T cell tolerance and anergy. In anergic T cells, the chromatin binding site of NR4A1 was consistent with the site of AP-1, a key transcription factor that regulates T cell activation. Next, it was found that NR4A1 can competitively bind to the binding site of AP-1 in chromatin, thereby suppressing the expression of target genes regulated by AP-1. At the same time, the NR4A1 binding site was positively correlated with the level of histone acetylation (H3K27ac) in the chromatin super-enhancer region. Overexpression of NR4A1 enhances the level of H3K27ac in this region, thereby up-regulating the transcription of immune tolerance and exhaustion-related genes; while knocking out NR4A1 down-regulates H3K27a at these sites. The above studies have identified a new transcriptional regulatory T cell tolerance and exhaustion mechanism with NR4A1 as the core. TTCF21 and GATA6 also performed conspicuously in this network. The transcription factor TCF21 is involved in the differentiation of mesenchymal cells into epithelial cells, and its abnormal methylation occurs in lung and head and neck tumors (29). NSCLC samples showed that the TCF21 promoter was hypermethylated and TCF21 protein expression was reduced. Multivariate analysis showed that TCF21 expression was lower than normal in both histological types (LUSC and LUAD) and was not correlated with gender, smoking, and EGFR mutation status. These results indicated that DNA methylation plays a key role in the development of lung tumors, and TCF21 may be a potential candidate methylation biomarker for early NSCLC screening (30). Previous studies calculated aberrantly expressed lncRNAs based on TCGA RNA-seq data, and found that GATA6-AS1 was of extremely high diagnostic value and played vital parts in the survival and development of LUSC. GATA6-AS1 was also closely related to the mitogen-activated protein kinase (MAPK) signaling pathway, which may be an important way for it to affect the prognosis of patients with LUSC (31, 32). However, these two transcription factors were rarely reported in tumor immunology, suggesting that epigenetic changes of TFs may affect immune gene expression and tumor characteristics to a certain extent. The TF-IRG regulatory networks we constructed will help to inform and guide future mechanism analysis.

To develop a manageable method to monitor the immune status of LUSC patients and predict clinical outcomes, we created a prognostic marker based on IRGs. Aramburu et al. combined alterations and expression of gene copy numbers, together with clinical parameters to establish a new comprehensive bioinformatics strategy as a more sophisticated prognostic approach (33). Since then, increased studies have begun to integrate immune-related genetic alterations and clinicopathologic parameters to predict survival. Recently, Xiaoshan S et al. established an IRGs prognostic risk model in lung adenocarcinoma based on TCGA data and validated it with GEO data. This study revealed the potential value of the IRGs model in improving TNM staging for survival predictions in LUAD (10). In our study, we filtered out 15 immune genes through multivariate analysis and calculated the immune gene Risk Index (IRI) based on these genes to stratify patients with LUSC and judge their prognosis. It is impressive that IRI can successfully distinguish patients, show excellent clinical feasibility, and perform moderately in clinical applicability and accuracy. Moreover, IRI can be used as an independent prognostic risk indicator and was positively correlated with patients' age.

Furthermore, IRI could be used not only as a prognostic marker but also as an indicator of immune status. The level of immune cell infiltration reflected by IRI enables us to adjust the treatment plan. In 1863, Virchow detected the infiltration of white blood cells in tumor tissues and proposed the relationship between inflammation and cancer. The importance of this cancer-related inflammation was gradually recognized. During the chronic inflammatory immune response, the complex interactions between tumor cells, immune cells, and inflammation gradually produce a tumor microenvironment that is conducive to angiogenesis and immunosuppression (34, 35). Eventually, tumor cells escape the control of the immune system and cancer occurs (36). Currently, the role of tumor-associated neutrophils (TANs) is still controversial, and neutrophils are the most abundant immune cell type in NSCLC (37). TANs may have high functional plasticity, which can not only fight cancer but also promote cancer in tumors (38). Previous studies have proposed that TANs have a two-sided phenotype similar to tumor-associated macrophages (TAMs), with both the “N1” type inhibiting tumor growth and the “N2” type promoting tumor growth and malignant metastasis (39). Then some studies proposed that TANs can maintain functional plasticity, that is, they can “alternately activate” when exposed to the tumor microenvironment. For example, the presence of transforming growth factor-β (TGF-β) promoted the proto-tumor phenotype (N2-TANs), while the presence of interferon-β (IFN-β) or the suppression of the TGF-β signaling pathway led to an anti-tumor (or N1) phenotype in TANs (39, 40). Our study found that IRI was positively correlated with the level of neutrophils infiltration, suggesting that the combination of existing PD-1 related therapy with TGF-β inhibition or IFN-β activation could be considered in the treatment of patients in the high-risk group, thereby exerting the potential antitumor effect of TANs. A strong cancer antigen presentation is a crucial step of the Cancer-Immunity Cycle, and also a necessary prerequisite for PD-1 blockade therapy to be effective (41). Dendritic cells (DCs) are the most powerful professional antigen-presenting cells (APCs) in the immune system, which play a key role in the initiation and regulation of immune response (42). In our study, the infiltration level of DCs was positively correlated with IRI. These results indicated that higher DCs might be observed in high-risk patients. Additionally, although there was no significant statistical difference in the infiltration of CD8 T cells, it was not difficult to find that there was a trend of higher levels of CD8 T infiltration in the high-risk groups. Based on the above results, we speculated that perhaps the high-risk group was more likely to benefit from immunotherapy. IRI may have the potential to serve as a predictor of increased immune cell infiltration and as a screening index for predominant populations for immunotherapy. Our research confirmed and expanded the discovery of IRGs that are essential for the treatment and prognosis of lung squamous cell carcinoma.

However, the present research still has several limitations and deficiencies. First of all, our study was only based on TCGA data and lacked validation with some other independent cohorts. Secondly, this study only analyzed the data in bioinformatics, and some of the hypotheses proposed need to be verified and discussed in subsequent *in vitro* and *in vivo* experiments. We also look forward to addressing these issues in future experiments.

## Conclusions

In summary, based on the TCGA-LUSC database and the ImmPort database, we have identified and verified the immune-related prognostic risk score, which can be used as an independent prognostic marker for evaluating the survival of patients with lung squamous cell carcinoma. Secondly, this score can partly reflect the tumor infiltration immune microenvironment status, which is closely related to the response rate of immunotherapy and can provide a reference for patients with lung squamous cell carcinoma, especially in advanced patients, to choose whether to immunotherapy.

## Data Availability Statement

Publicly available datasets were analyzed in this study, these can be found in The Cancer Genome Atlas (https://portal.gdc.cancer.gov/).

## Author Contributions

YZ and GL: conception, design, and manuscript writing. YZ and SL: collection and assembly of data. YZ and CL: data analysis and interpretation. All authors: paper revision and final approval of manuscript.

## Conflict of Interest

The authors declare that the research was conducted in the absence of any commercial or financial relationships that could be construed as a potential conflict of interest.

## Abbreviations

AJCC: American joint committee on cancer
APCs: antigen-presenting cells
AUC: the area under the curve
BP: biological process
CC: cellular component
DCs: dendritic cells
FDR: false discovery rate
GO: gene ontology
IFN-β: interferon-β
IRGs: immune-related genes
IRI: immune gene risk index
KEGG: kyoto encyclopedia of genes and genomes
LUAD: lung adenocarcinoma
LUSC: lung squamous cell carcinoma
MF: molecular function
NSCLC: non-small cell lung cancer
OS: overall survival
p-IRGs: prognosis-associated IRGs
r-IRGs: risk immune-related genes
TCGA: the cancer genome atlas
TAMs: tumor-associated macrophages
TANs: tumor-associated neutrophils
TFs: transcription factors
TGF-β: transforming growth factor-β
TME: tumor microenvironment
TPS: tumor proportion score.
